# In this Issue

**DOI:** 10.1111/cas.14948

**Published:** 2022-03-06

## An aging‐related signature predicts favorable outcome and immunogenicity in lung adenocarcinoma

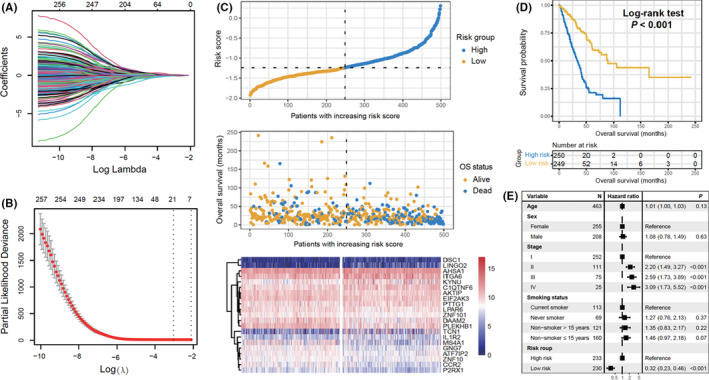

Aging is a well known risk factors for many diseases and cancer is no different. It is difficult to determine the exact mechanism of how aging contributes to tumorigenesis and response to treatment, but studies have used transcriptomic analysis of large populations to determine aging‐related genes. In this study, Zhang et al used a similar transcriptomic approach to generate an aging risk signature composed of 21 aging‐related genes. They used this aging risk signature to analyze immune infiltration and tumor immunogenicity, factors associated with good response to immune checkpoint inhibitors (ICIs), as well as survival status. In lung adenocarcinoma (LUAD) cohorts, they were able to validate that their aging signature was an independent prognostic factor. The mechanism of how this aging risk signature affects survival and ICI response remains unclear, but further research may lead to improved care for LUAD patients.

https://onlinelibrary.wiley.com/doi/10.1111/cas.15254

## MEK inhibition suppresses metastatic progression of KRAS‐mutated gastric cancer

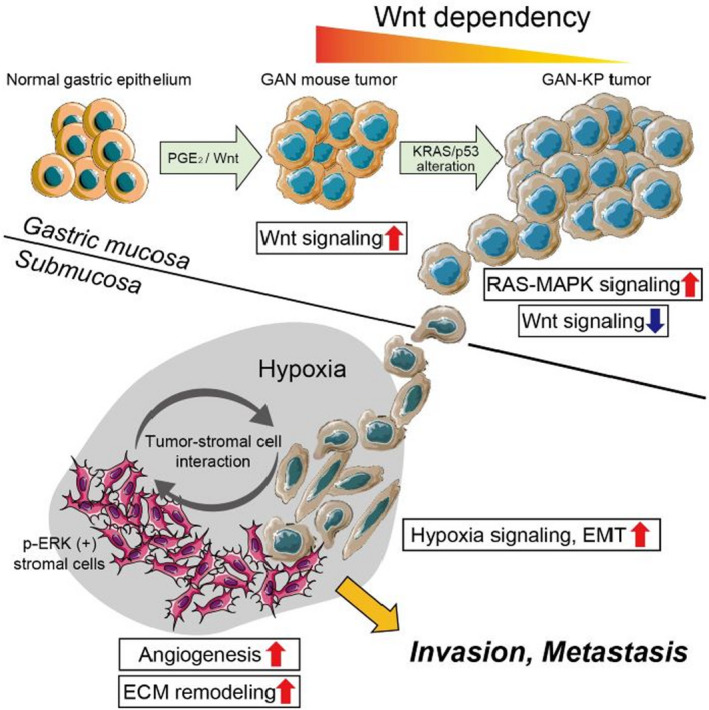

Gastric cancer is especially prevalent in East Asia and a majority of patients present with locally advanced disease, which is associated with a poor prognosis. As there are no optimal screening strategies, therapeutics that can stop the metastatic progression of gastric cancer are sorely needed. In this study, Yamasaki et al generated an organoid based model of gastric cancer to study the mechanism of metastatic progression and potential therapies. By utilizing spatial transcriptomics, they identified stromal cells at the tumor invasive front that were positive for phosphorylated extracellular signal‐regulated kinase (ERK). This suggested that the hypoxia induced signaling in this tumor microenvironment was driven by mitogen‐activated protein kinase (MAPK) signaling. They found that trametinib, a MEK (MAPK kinase) inhibitor, suppressed the formation of gastric tumors and metastatic spread in their gastric cancer model. This suggests that trametinib may be an effective therapeutic for locally advanced gastric cancer.

https://onlinelibrary.wiley.com/doi/10.1111/cas.15244

## Enforced dual‐specificity tyrosine‐regulated kinase 2 expression by adenovirus‐mediated gene transfer inhibits tumor growth and metastasis of colorectal cancer

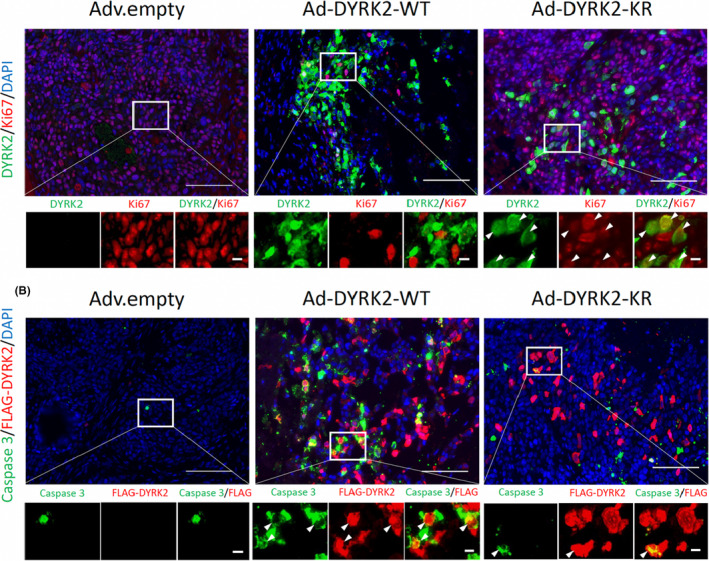

Colorectal cancer (CRC) is a leading cause of cancer death in both men and women. Screening colonoscopies have significantly improved survival, but many patients still present with advanced disease. Only a small portion of patients with metastatic disease have microsatellite instability‐high CRC that responds to immune therapy so novel therapeutics need to be developed. In this study, Imaizumi et al examined the use of adenovirus vectors (Advs) for gene therapy. Previous studies have shown that dual‐specificity tyrosine‐regulated kinase 2 (DYRK2) induces apoptosis in response to DNA damage and is downregulated in CRC. The group generated Advs that forced expression of DYRK2, which inhibited colon cancer growth in vivo and vitro. Further development of the Advs may make gene therapy viable for the treatment of cancer.

https://onlinelibrary.wiley.com/doi/10.1111/cas.15247

